# Biotinidase Deficiency With Suspected Sotos Syndrome: A Rare Entity

**DOI:** 10.7759/cureus.8000

**Published:** 2020-05-07

**Authors:** Sumyya Ghazal, Aiman Ali, Taha Bin Arif, Fatima Memon, Laraib Malik

**Affiliations:** 1 Pediatrics, Abbasi Shaheed Hospital, Karachi, PAK; 2 Internal Medicine, Dow University of Health Sciences, Karachi, PAK

**Keywords:** biotinidase, biotin, metabolic, sotos syndrome, genetic

## Abstract

Biotinidase deficiency (BTD) is a rare yet treatable metabolic autosomal recessive (AR) disorder in which the body is unable to recycle the vitamin biotin. Early diagnosis and treatment can be life-saving, but some symptoms of the disease are irreversible, and the condition can even prove to be fatal if not correctly diagnosed and managed. Here we present a case of a six-month-old child who presented with cough, fever, and difficulty in breathing. Respiratory examination revealed deep subcostal and intercostal recessions, bilateral crepitations, and wheezes. On central nervous system (CNS) examination, the baby had a low Glasgow Coma Scale (GCS) score of 10 while the tone was decreased, and bulk was increased in all four limbs. Chest X-ray revealed haziness at the right middle and lower lobes. Antibiotics were started keeping pneumonia, bronchiolitis, and sepsis in mind along with an initial diagnosis of inborn error of metabolism (IEM). As the patient's condition deteriorated, nasal bubble continuous positive airway pressure (CPAP) and nebulization were provided and later put on a ventilator. Arterial blood gases (ABGs) showed severe metabolic acidosis and compensatory respiratory alkalosis with an anion gap of 15. Urine profile for organic acid was performed, and the diagnosis of sepsis with BTD was made. Unfortunately, our patient expired on the fourth day of admission before a biotin injection could be searched and administered. Moreover, our patient was also suspected of a possible Sotos syndrome, which is a rare genetic disorder characterized by excessive growth in the initial years of life. The case highlights the significance of the diagnosis of such metabolic disorders in the natal period of life and their immediate management.

## Introduction

Biotinidase deficiency (BTD) is an autosomal recessive (AR) neurocutaneous metabolic disorder resulting in the impaired release of biotin, a vitamin, and an essential coenzyme in many carboxylation reactions. It is a rare disease with an approximated incidence of 1 per 60,089 newborns [1]. BTD deficiency can be classified as profound, which is characterized by less than 10% of normal serum enzyme activity and partial, where mean enzyme activity is 10%-30% of normal [1]. Most frequent initial neurological symptoms include seizures, usually myoclonic [2]. Hypotonia, ataxia, skin rash, hyperventilation, developmental delay, optic atrophy, ketoacidosis, organic aciduria, and hyperammonemia are some other features with which patients present [2-3]. Partial BTD deficiency usually has minimum or no symptoms [4]. Whereas profound BTD deficiency is a more severe form which if untreated, in some individuals, can cause severe metabolic derangements that can lead to coma or even death [5]. Clinical and biochemical variability is seen among patients with impaired BTD activity [6]. Treatment with biotin can reverse many symptoms, with the exception of developmental delay, optic atrophy, and hearing loss [3].

The rarity of BTD deficiency and delay in its diagnosis due to lack of neonatal screening and various clinical presentations make this case an interesting one to report. Furthermore, suspicion of Sotos syndrome has embellished this case. Sotos syndrome or cerebral gigantism, is a rare multi-systemic genetic disorder. The diagnosis is usually made clinically when the patient presents with distinctive facial features (broad forehead, macrocephaly, hypertelorism, flat-bridged nose, and large ears), large stature, psychomotor delay, and speech impairment [7]. It is not a life-threatening disorder, and symptoms may resolve. Here we report a case of a six-month-old male who was brought to the pediatric emergency department (PED) with complaints of cough, difficulty in breathing, and fever.

## Case presentation

A six-month-old male infant, vaccinated to date, weighing 11 kg, was brought to PED at Abbasi Shaheed Hospital, Karachi, with complaints of cough, difficulty in breathing, and fever for four days. The cough was productive and was accompanied by rapidly progressive difficulty in breathing. The patient developed high-grade fever (102°F-104°F) with rigors, which gradually subsided. He also had an episode of self-aborting fits lasting for a few seconds. He was the first product of a nonconsanguineous marriage and was born via emergency cesarean (C-section) at term. No family history of miscarriage, stillbirth, death at an early age or any chronic disease was present. Birth history was also unremarkable. Developmental milestones were appropriate for age until four months, but regression was noticed during episodic fits two months ago. There was no positive family history of convulsions.

**Figure 1:**
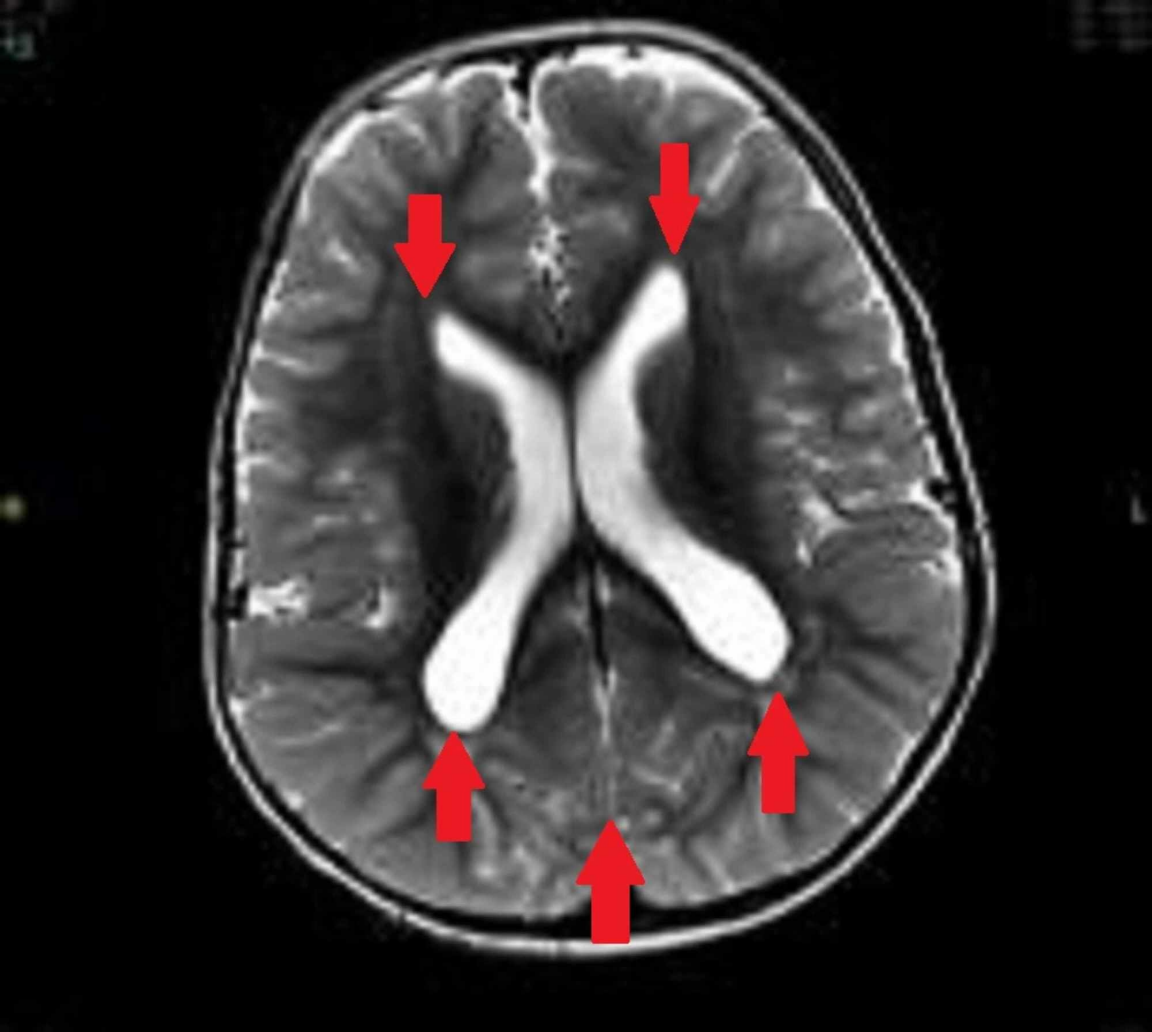
CT scan brain showing megalencephaly with enlarged ventricles and prominent trigone (red arrows).

Past history revealed episodes of afebrile fits at four months of age, which were generalized tonic-clonic in nature. These seizures continued for three weeks. As the episodes were self-limiting, the parents did not seek medical care at first, but the patient lost his milestones (neck holding, sitting with support) as a consequence of the continued fits. He was then taken to a private hospital where complete workup was done. He was suspected of some metabolic disorder considering an anion gap of 22 and acidic urine sample with 4+ ketone bodies and raised levels of ammonia. CT scan brain showed generalized enlarged brain parenchyma (megalencephaly) with prominent trigone and enlarged prominent ventricles (Figure 1). He was advised for MRI brain, which was not performed. Leveteracitem was administered during his stay. The patient had a dramatic growth spurt with rapidly increasing weight and regression of achieved milestones in the previous two months.

General physical examination (GPE) concluded a malnourished, pale-looking, floppy baby with severe respiratory distress. Some dysmorphic features such as abnormally tall stature, relatively large head size and limbs, broad forehead, and hypertelorism were also observed. Anthropometric measurements were greater than or equal to the 97th centile, i.e. fronto-occipital circumference of 46 cm, length of 75 cm, and weight of 11 kg. The baby was afebrile with a heart rate (HR) of 140 beats/min, respiratory rate (RR) of 72 breaths/min, blood pressure (BP) of 90/60 mmHg, and oxygen saturation (OS) of 85% at room air and 92% at 10L nasal bubble continuous positive airway pressure (CPAP). On respiratory examination, the child had deep subcostal and intercostal recessions with the use of accessory muscles. Percussion note was resonant equally in all lung fields. However, bilateral crepitations and wheezes were heard all over the chest, more in the middle and lower chest. Central nervous system (CNS) examination revealed a low Glasgow Coma Scale (GCS) score of 10 with decreased tone and increased bulk in all four limbs and a diminished gag reflex. The rest of the examinations were insignificant.

**Figure 2:**
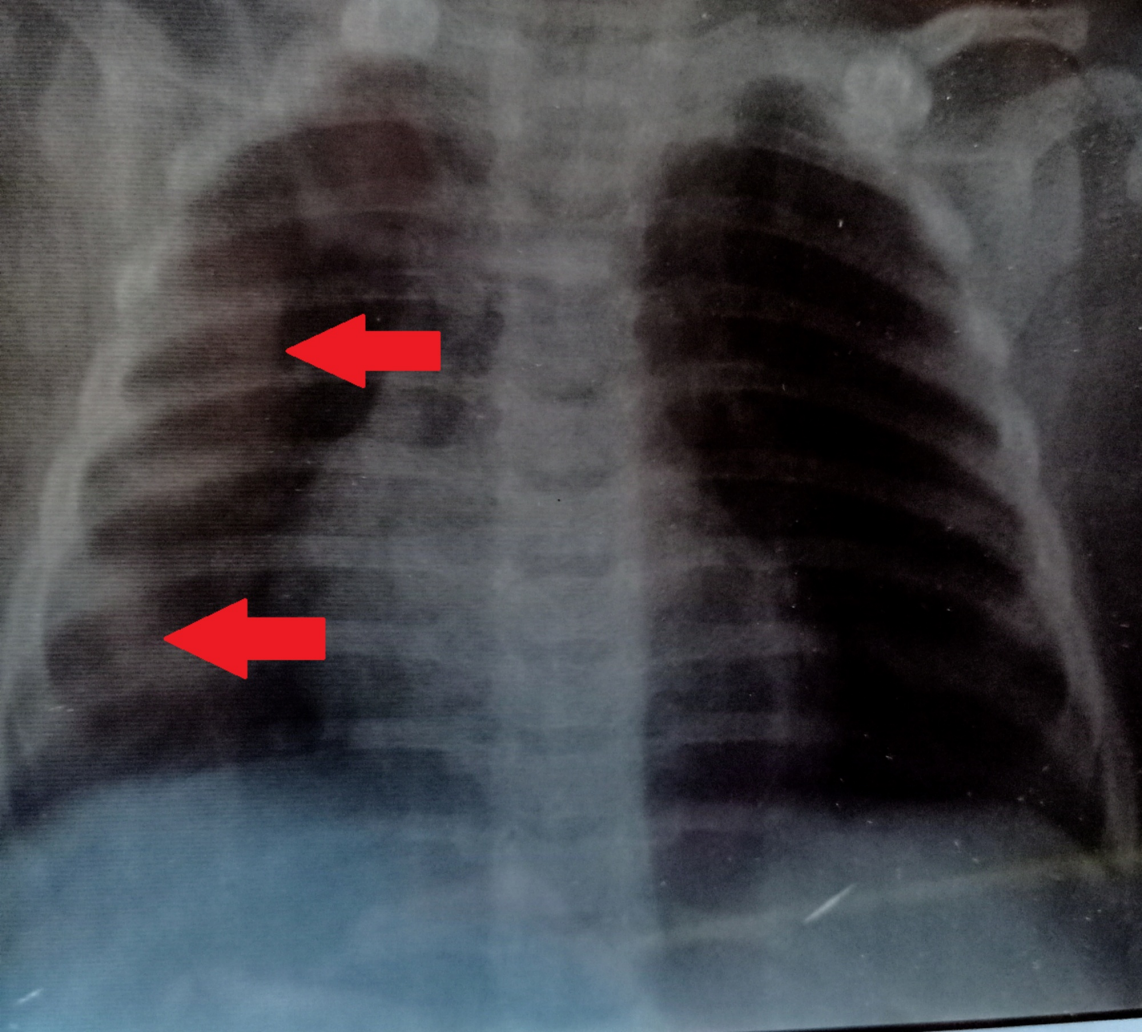
Chest X-ray revealing haziness in the right middle and lower lobes (red arrows).

Differential diagnosis of inborn error of metabolism (IEM) with pneumonia, bronchiolitis, or sepsis was established. The initial laboratory investigations (at the day of admission) revealed a hemoglobin (Hb) level of 11 g/dL, hematocrit of 32.2%, raised total leukocyte count (TLC) of 14.6 x 109/L, and platelet count (PLT) of 276 x 109/L. Renal function tests (RFTs) and coagulation profile were normal. The patient was initially kept nil per oral (NPO), and regular charting of his vitals was carried out. He was nebulized with normal saline, ipratropium, and salbutamol four hourly and was managed on intravenous (IV) ceftriaxone, vancomycin, and hydrocortisone eight hourly. The patient's condition deteriorated on the second day, and nasal bubble CPAP and nebulization were given back to back. Hydrocortisone and ceftriaxone were stopped while vancomycin was continued, and ceftazidime was added in the regimen. During his stay, the patient developed seizures for which phenytoin was started. Crepitations were also heard all over his chest, for which he was administered with IV furosemide. Chest X-ray revealed haziness at the right middle and lower lobes. The left lung appeared normal, with no mediastinal shift (Figure 2).

Laboratory investigations were repeated on the third day, and a slight decrease in hemoglobin (7.7 g/dL), hematocrit (24%), and platelets (240 x109/L) was observed. Serum electrolytes revealed hypernatremia [sodium (Na)= 163 mEq/L, N=135-146], hypokalemia [potassium (K)= 2.8 mEq/L, N=3.5-5.5], and hyperchloremia [chloride (Cl)= 135 mEq/L, N=95-108]. Arterial blood gases (ABGs) showed severe chronic metabolic acidosis and compensatory respiratory alkalosis with an anion gap of 15. Blood urea nitrogen (BUN) was elevated while creatinine was normal. The initial blood culture report was positive for Gram-positive and Gram-negative rods. Due to labored breathing, OS began to drop for which he was put on a ventilator. Packed cell volume (PCV) and fresh frozen plasma (FFP) were transfused, and inotropic support was started. Laboratory investigations on days 1 and 3 are summarized in Table 1.

**Table 1:** Laboratory investigations of the patient during the hospital stay. Hb, hemoglobin; Ht, hematocrit; TLC, total leukocyte count; PLT, platelets; BUN, blood urea nitrogen; Cr, creatinine; Na, sodium; K, potassium; Cl, chloride; HCO3, bicarbonate; Ca, calcium; PT, prothrombin time; APTT, activated partial thromboplastin time; INR, international normalized ratio

Laboratory investigation	Normal value (units)	Day 1	Day 3
Hb	10.5-14.1 (g/dL)	11	7.7
Ht	33-43 (%)	32.2	24
TLC	6.0-17 (x10^3^/uL)	14.6	14
PLT	150-400 (x10^3^/uL)	276	240
BUN	11.3-14.1 (mg/dL)	15	32
Cr	0.3-0.7 (mg/dL)	0.47	0.7
Na	135-146 (mEq/L)	145	163
K	3.5-5.5 (mEq/L)	3.7	2.8
Cl	95-108 (mEq/L)	117	135
HCO3	19-28 (mEq/L)	16	13
Ca	8.8-10.8 (mg/dL)	9.5	7.9
PT	10-12 (seconds)	6	7
APTT	28-45 (seconds)	30	35
INR	<1.1	0.9	1.0
Anion gap	8-12	12	15

On the fourth day, no improvement in patients' condition was seen despite ventilatory support. ABGs were repeated, which revealed a more chronic hypocapnic picture. Urine profile for organic acid by gas chromatography-mass spectrometry (GC-MS) was performed, which was suggestive of biotin responsive multiple carboxylase (biotinidase or holocarboxylase synthetase) deficiency, and a definitive diagnosis of sepsis with biotinidase deficiency (BTD) was established. However, a delay in diagnosis and financial constraint became a hindrance in the timely administration of biotin injection, which resulted in the aggravation of symptoms. Unfortunately, the baby expired on the fourth day of admission. The child was also suspected of a possible Sotos syndrome considering dysmorphic features (abnormally tall stature, relatively large head size and limbs, broad forehead, and hypertelorism). Moreover, his anthropometric measurements were above the 97th centile. However, due to the deteriorating condition of the child, no genetic testing could be performed to confirm the diagnosis of Sotos syndrome.

## Discussion

Several metabolic enzymes (acetyl CoA carboxylase, pyruvate carboxylase, propionyl CoA carboxylase, and 3-methyl cronotonyl carboxylase) require a unique cofactor called biotin (vitamin B7). It functions as a carbon dioxide donor in carboxylation reactions by attaching to the apocarboxylases. Holocarboxylase synthetase is an enzyme that catalyzes the biotinylation of apocarboxylases. This reaction depends on adenosine triphosphate (ATP) and results in the formation of an intermediate, biotinyl adenosine monophosphate. The turnover of carboxylases produces biotinyllysine from which biotin is rejuvenated by the action of an enzyme known as BTD [8]. BTD is a specific amidolyase that is also required for the production of dietary protein-bound biotin. The deficiency of this enzyme is inherited as AR neurometabolic disorder that can lead to behavioral changes, lack of coordination, learning disabilities, and seizures. Although this disorder is infrequent, the combined incidence of partial and profound BTD deficiency is estimated to be about 1:13,909 live births in Brazil. This prevalence is much higher than the incidence rates reported in other populations worldwide [9].

Mutations in the BTD gene cause BTD deficiency. This gene is located at chromosome 3p25 locus and has four exons of length 79, 265, 150, and 1502 base pairs (bp), respectively. About 61 different mutations in three of the four exons have been identified to cause BTD deficiency [10]. Clinical manifestations of BTD deficiency vary with the severity of deficiency of enzyme. Partial BTD deficiency (10%-30% enzyme activity) presents with milder symptoms, especially during stress, such as infection. However, it may be asymptomatic or present solely as intractable seborrhoeic dermatitis and can be resolved with biotin supplementation. Profound deficiency (less than 10% enzyme activity) can present as early as one week of age with neurological manifestations (neurodevelopmental delay, metabolic encephalopathy, muscular hypotonia, and drug-resistant seizures), cutaneous manifestations (alopecia, eczema, or seborrhoeic dermatitis), respiratory issues (apnea, hyperventilation, and stridor), and immunodeficiency syndromes from T-cell abnormalities [11]. Our patient presented with atypical symptoms of fever, cough, and difficulty in breathing. However, history revealed episodes of self-limiting afebrile seizures. Also, a low GCS with a diminished gag reflex, muscular hypotonia, and severe respiratory distress with deep subcostal and intercostal recessions were observed on clinical examination.

According to the American College of genetics and genomics (ACMG) guidelines, neonatal screening and confirmatory diagnosis of BTD deficiency comprise both enzymatic and molecular testing approaches [12]. The most common cause of decreased enzymatic activity is sample mishandling in laboratories. Unless these variables are adequately controlled, a definitive diagnosis of decreased enzymatic activity cannot be established. The optimal method of determining enzymatic activity is to collect the patient's sample with a sample from normal control (unrelated individual) and both parents simultaneously [12]. BTD deficiency can be confirmed by DNA analysis by either full gene sequencing or allele targeted methods [13]. Considering the initial presentation, past medical records, and clinical examination findings, our patient was suspected of an IEM with pneumonia, bronchiolitis, or sepsis. Adequate respiratory support with nebulization and antibiotics was provided, but no improvement in the patient's condition was observed. A significant bacterial growth on blood culture and elevated BUN with severe metabolic disturbances strongly pointed towards sepsis. However, there was no amelioration in symptoms and ABGs despite changing antimicrobial regimen and providing ventilatory support. Considering an IEM, a thorough literature search was done, and the child's urine was tested for BTD levels, which revealed a significant deficiency of enzyme. Newborn screening programs in many countries do not include BTD deficiency in their panel of screened conditions [13]. This can be an essential reason for the delay in the diagnosis of such rare conditions with poor mortality rates, as demonstrated by our case.

Treatment of BTD deficiency is oral free biotin (vitamin H, coenzyme R, or part of the vitamin B complex), typically prescribed as an initial dose of 5-10 mg/dL. Bound biotin manufactured as multivitamin supplements does not treat BTD deficiency. Treatment should commence once the diagnosis is finalized. Symptoms of the disorder may disappear with biotin supplementation. However, some clinical features like developmental delay, optic atrophy, and diminished hearing are irreversible once they occur [14]. The patient has to take these supplements for his or her entire life. Furthermore, genetic counseling is required for families of a child with BTD deficiency. Our patient could not be started on biotin supplementation due to a delay in definitive diagnosis and continuous rapid deterioration of symptoms.

**Figure 3:**
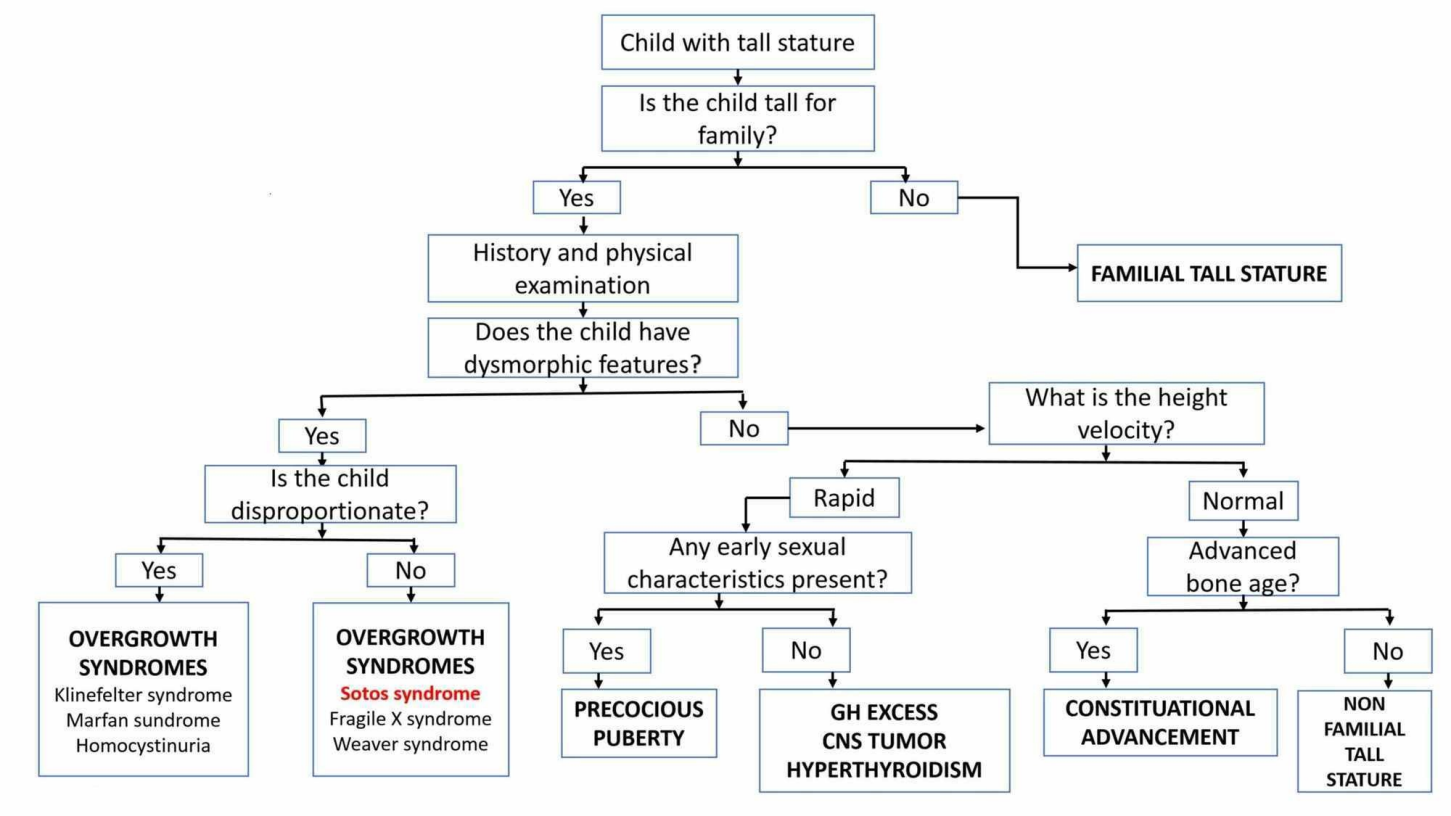
Algorithm for evaluating a child with tall stature. Adapted from Refs. [16-17] GH, growth hormone; CNS, central nervous system

General physical examination of our patient revealed some unusual anthropometric measurements and dysmorphic features, which highly indicated towards Sotos syndrome. Sotos syndrome is a genetic disorder characterized by excessive growth before and after birth, a dilochocephalic head, distinctive facial features, and a nonprogressive neurological disorder with intellectual disability. It has a reported incidence of 1 in 10,000 to 14,000 neonates [15]. It is caused by mutations in the nuclear receptor-binding SET domain protein 1 (NSD1) gene. As there is no biomarker of Sotos syndrome, diagnosis is usually based on clinical grounds by excluding other common causes of tall stature, as demonstrated in the algorithm (Figure 3) adapted from studies by Meazza et al. and Stalman et al. [16-17]. It can be further confirmed by DNA analysis by fluorescence in situ hybridization (FISH) or multiplex ligation-dependent probe amplification (MLPA) technique to detect chromosome 5q35 microdeletions and partial NSD1 gene deletions [18]. Our patient was highly suspected of Sotos syndrome based on clinical findings (anthropometric measurements greater than 97th centile, large head and limbs, distinct facial features, developmental delay with diminished milestones, and neurological abnormalities). However, a definitive diagnosis of Sotos syndrome through molecular genetic analysis could not be established in our case due to financial constraints and immediate worsening of symptoms secondary to sepsis with BTD deficiency.

## Conclusions

There is often a delay in the diagnosis and management of various metabolic disorders due to their low incidence and significant overlap of symptoms with other diseases of the neonatal period, which may result in unfavorable circumstances. Although BTD deficiency is a treatable condition, delayed diagnosis and failure of the timely administration of biotin led to the death of our patient despite constant supportive efforts. Lack of newborn screening programs for BTD deficiency and other inborn errors of metabolism in developing countries plays a pivotal role in increasing the neonatal mortality rate due to such disorders.
